# Precursor-Concentration-Regulated CuO-CeO_2_ Catalysts with Tailored Oxygen Species and Redox Behavior for Efficient C_3_H_6_ Catalytic Combustion

**DOI:** 10.3390/molecules31203653

**Published:** 2026-10-13

**Authors:** Shuo Zhang, Tianze Zhang, Yuxiao Lu, Shiya Meng, Zhenkun Sun, Yuanqiang Duan

**Affiliations:** Key Laboratory of Energy Thermal Conversion and Control, Ministry of Education, School of Energy and Environment, Southeast University, Nanjing 210096, China

**Keywords:** precursor-concentration engineering, self-sustained flame pyrolysis, CuO-CeO_2_ mixed oxides, redox behavior, C_3_H_6_ catalytic combustion

## Abstract

Flame synthesis is a promising route for producing mixed-oxide catalysts, but the role of precursor concentration in regulating catalyst formation during self-sustained flame pyrolysis remains insufficiently understood. In this work, CuO-CeO_2_ mixed-oxide catalysts were prepared by varying the total metallic precursor concentration from 0.04 to 0.28 mol·L^−1^ while keeping the Cu/(Cu + Ce) molar ratio fixed at 35 mol.%. This strategy enables the influence of precursor concentration to be distinguished from that of metal composition and provides insight into the formation of CuO-CeO_2_ catalysts under a self-sustained flame environment. The results show that precursor concentration strongly affects particle aggregation, crystallite growth, Cu species distribution and possible lattice incorporation, oxygen species distribution, and low-temperature reducibility. Among the prepared catalysts, CuCe0.20 exhibits the most favorable balance among textural characteristics, Cu-Ce interaction, oxygen species distribution and redox behavior, and surface reactive oxygen availability. As a result, CuCe0.20 achieves 90% C_3_H_6_ conversion at 380 °C and nearly complete conversion at 450 °C. More importantly, it maintains stable activity during a 600 min test at 450 °C with an apparent CO_2_ selectivity of 99.9%. Post-reaction characterization further reveals that the CeO_2_ framework and Cu-Ce interaction are largely retained, while the relative contribution of lattice oxygen increases after long-term reaction, accompanied by retained low-temperature reducibility. This work extends the design of flame-made CuO-CeO_2_ catalysts from composition optimization to precursor-concentration engineering and demonstrates a practical strategy for preparing stable non-noble metal catalysts for VOC catalytic combustion.

## 1. Introduction

Typical volatile organic compounds (VOCs), such as C_3_H_3_N, CH_3_CHO, and C_3_H_6_, can irritate the nervous system, skin, and eyes, and may cause organ toxicity in humans and animals. Some aldehydes and ketones also contribute to photochemical haze, adversely affecting plant growth [1]. In the atmosphere, VOCs react with hydroxyl radicals to form free-radical intermediates, such as alkyl peroxides, which promote the conversion of NO to NO_2_ [2,3]. Their oxidation products can further participate in secondary organic aerosol (SOA) formation, increasing PM_2.5_ concentrations [4]. Among these VOCs, C_3_H_6_ removal has attracted considerable attention due to its large industrial emissions and the high energy consumption required for complete elimination [5,6].

To address this challenge, catalytic combustion is a promising strategy for VOC removal because of its relatively low operating temperature, reduced energy consumption, and high conversion efficiency [7]. Therefore, developing catalysts with high activity, good stability, and low cost is essential for efficient C_3_H_6_ combustion. Transition metal oxides have been widely explored for this purpose. For example, Ma et al. [8] reported that Co_3_O_4_ with a specific surface area of 99.7 m^2^·g^−1^ achieved complete C_3_H_6_ conversion at 260 °C, while Assebban et al. [9] obtained 90% C_3_H_6_ conversion at 339 °C over Co_3_O_4_-coated natural clay honeycomb monoliths. CuO-CeO_2_ mixed oxides are also well-known oxidation catalysts for CO preferential oxidation, water-gas shift, methanol oxidative steam reforming, and soot oxidation reactions [10,11,12,13,14,15,16]. Their abundant solid-solution structures endow them with favorable catalytic activity and stability. Compared with their extensive applications in CO preferential oxidation, water-gas shift, methanol oxidative steam reforming, and soot oxidation reactions, the process-oriented regulation of CuO-CeO_2_ catalysts for C_3_H_6_ catalytic combustion remains insufficiently understood.

The catalytic performance of CuO-CeO_2_ mixed oxides is strongly governed by their physicochemical properties, including crystallite size, specific surface area, adsorbed oxygen species, redox ability, oxygen vacancies, and oxygen mobility, all of which are closely related to the synthesis method and preparation parameters [10]. Various approaches, such as impregnation, co-precipitation, hydrothermal synthesis, urea-nitrate combustion, hard-template methods, and flame spray pyrolysis (FSP), have been employed to prepare CuO-CeO_2_ catalysts [17,18,19,20,21,22,23,24,25]. Yang et al. [26] showed that nitrate thermal decomposition produced CuO-CeO_2_ catalysts with the highest surface area, smallest particle size, strongest Cu-Ce interaction, and best Cu dispersion, leading to superior CH_4_ combustion activity. Among these methods, FSP is particularly attractive because it promotes strong Cu-Ce interactions, generates small particles with controllable morphology and highly dispersed species, and enables rapid catalyst production suitable for scale-up [27,28,29,30,31].

Although flame-synthesized CuO-CeO_2_ catalysts have been explored for C_3_H_6_ catalytic combustion, existing studies, including our previous work [31], mainly focused on the effect of Cu loading on solid-solution formation and catalytic activity. In such composition-regulation strategies, changes in the Cu/Ce ratio simultaneously affect catalyst composition, Cu dispersion, and particle-formation behavior, making it difficult to identify the independent role of precursor concentration during flame synthesis. However, precursor concentration is a critical process parameter in self-sustained flame pyrolysis (SSFP) because it directly influences droplet solute loading, particle nucleation density, collision frequency, aggregation behavior, and the final distribution of Cu species in the CeO_2_ matrix.

Therefore, in this work, the Cu/(Cu + Ce) molar ratio was fixed at 35 mol.% and the total metallic precursor concentration was systematically varied from 0.04 to 0.28 mol·L^−1^. This design enables independent evaluation of precursor-concentration effects on microstructure, oxygen-species distribution, redox properties, catalytic performance, and post-reaction stability of CuO-CeO_2_ catalysts. Compared with previous composition-focused studies, this work provides a process-oriented understanding of SSFP catalyst formation and highlights precursor concentration as an effective parameter for tuning high-performance non-noble-metal catalysts for VOC catalytic combustion.

## 2. Experimental and Methods

**Figure 1:**
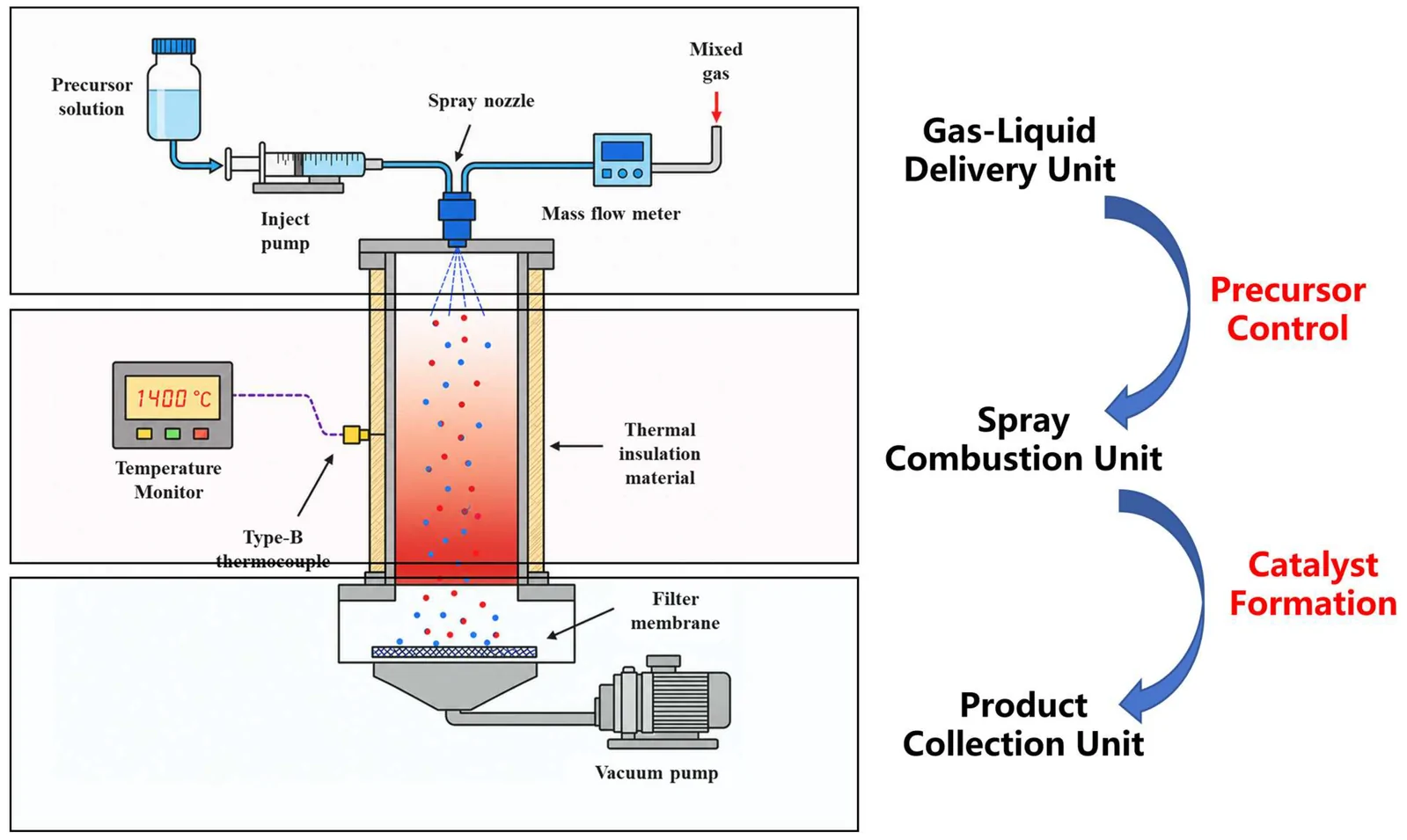
Schematic diagram of the self-sustained flame pyrolysis (SSFP) system used for catalyst synthesis.

The schematic diagram of the self-sustained flame pyrolysis (SSFP) system used for catalyst synthesis is shown in Figure 1. CuO-CeO_2_ mixed-oxide catalysts were synthesized by self-sustained flame pyrolysis (SSFP) using a previously developed flame synthesis platform [31]. To distinguish the effect of precursor concentration from that of metal composition, the Cu/(Cu + Ce) molar ratio was fixed at 35 mol.% while the total metallic precursor concentration was varied as 0.04, 0.12, 0.20, and 0.28 mol·L^−1^. Copper nitrate trihydrate (Cu(NO_3_)_2_·3H_2_O, AR, Nanjing Reagent, Nanjing, China) and cerium nitrate hexahydrate (Ce(NO_3_)_3_·6H_2_O, AR, Nanjing Reagent, Nanjing, China) were dissolved in ethanol, which served as both the solvent and fuel for sustaining the flame. During synthesis, the precursor solution was fed into the atomizing nozzle at 1.5 mL·min^−1^, and oxygen was supplied at 12 L·min^−1^ as the atomizing, carrier, and combustion-supporting gas. The atomized precursor solution was initially ignited with an external igniter, after which the flame was sustained by ethanol combustion without an additional pilot flame or auxiliary fuel. The generated particles were collected on a micro/nano filter membrane and denoted as CuCeX, where X represents the total metallic precursor concentration. Under the fixed Cu loading and gas-liquid feeding conditions, the structural and catalytic differences among the samples mainly reflect precursor-concentration-induced variations in droplet solute loading, particle aggregation, Cu species distribution, and oxygen species formation.

The prepared catalysts were characterized by X-ray diffraction (XRD, SmartLab, Rigaku, Akishima, Japan), scanning electron microscopy (SEM, S-4800, Hitachi, Tokyo, Japan), N_2_ adsorption-desorption (Micromeritics ASAP 2020 Plus HD88, Norcross, GA, USA), X-ray photoelectron spectroscopy (XPS, Thermo ESCALAB 250, Waltham, MA, USA), and H_2_ temperature-programmed reduction (H_2_-TPR). XRD patterns were recorded over 10–80° with a step size of 0.02°. N_2_ adsorption-desorption measurements were conducted at 77 K, and the specific surface area, pore size distribution, and pore volume were calculated using the BET method, BJH model, and adsorption data at P/P_0_ > 0.995, respectively. H_2_-TPR was performed by heating 50 mg of catalyst from 50 to 600 °C at 10 °C·min^−1^ under 5 vol.% H_2_/Ar at a total flow rate of 25 mL·min^−1^.

The catalytic combustion of C_3_H_6_ was evaluated in a vertical fixed-bed reactor. In each test, 300 mg of catalyst was placed in the middle of a stainless-steel reactor tube and supported by quartz wool. The feed gas contained 1 vol.% C_3_H_6_, 10 vol.% O_2_, and 89 vol.% N_2_, with a total flow rate of 900 mL·min^−1^, corresponding to a catalyst-mass-normalized volumetric feed rate of 180,000 mL g^−1^ h^−1^. The volumetric flow rates used for molar-flow calculations were referenced to 25 °C and 1 atm. The reactor temperature was increased at 5 °C·min^−1^, and the outlet gas was analyzed online using an FTIR gas analyzer after water removal by a silica gel dryer to minimize interference from water vapor. Although the silica gel may potentially retain certain polar partial oxidation byproducts, this possibility represents a limitation of the product analysis.

$$\mathrm{Conversion}\;(\%)=\frac{C_{{C_{3}H}_{6},in}-C_{{C_{3}H}_{6},out}}{C_{{C_{3}H}_{6},in}}\times 100\%$$

where $C_{{C_{3}H}_{6},in}$ and $C_{{C_{3}H}_{6},out}$ are the inlet and outlet concentrations of C_3_H_6_, respectively. To further verify the practical stability of the optimized catalyst, a long-term test was performed at 450 °C for 600 min. The spent catalyst was subsequently collected and characterized to clarify the post-reaction evolution of textural characteristics, crystalline phase, surface oxygen species, and redox properties. It should be noted that the concentration-based conversion represents an approximation because the total gas flow may change during C_3_H_6_ combustion. Under the present dilute feed conditions (1 vol.% C_3_H_6_ and 89 vol.% N_2_), the change in total dry-gas flow is limited. Assuming complete C_3_H_6_ oxidation and complete removal of the generated water, the maximum decrease in total dry-gas flow is 2.5%, corresponding to a theoretical deviation of less than 0.64 percentage points between the concentration-based and molar-flow-based conversions. Therefore, the concentration-based approximation is used here for comparative evaluation of catalytic activities under identical experimental conditions. An apparent CO_2_ selectivity was estimated from the measured dry-gas concentrations according to the following equation:

$$S_{{CO}_{2}}(\%)=\frac{C_{{CO}_{2},out}-C_{{CO}_{2},in}}{3(C_{{C_{3}H}_{6},in}-C_{{C_{3}H}_{6},out})}\times 100\%$$

where $C_{{CO}_{2},in}$ and $C_{{CO}_{2},out}$ are the inlet and outlet CO_2_ concentrations, respectively. The factor of 3 accounts for the three carbon atoms in each C_3_H_6_ molecule. For the quantitative evaluation of catalytic performance, the mass-specific C_3_H_6_ consumption rate was estimated as follows:

$$r_{{C_{3}H}_{6}}=\frac{F_{C_{3}H_{6},in}X_{{C_{3}H}_{6}}}{m_{cat}}$$

where $F_{C_{3}H_{6},in}$ is the inlet molar flow rate of C_3_H_6_, $X_{{C_{3}H}_{6}}$ is the fractional C_3_H_6_ conversion, and $m_{cat}$ is the catalyst mass.

## 3. Results and Discussion

### 3.1. Phase Composition, Surface Chemical States, and Redox Behavior

To establish the phase and surface-chemical basis for interpreting the subsequent textural and morphological evolution, the XRD and XPS results are discussed first, followed by the H_2_-TPR analysis of redox behavior.

**Figure 2:**
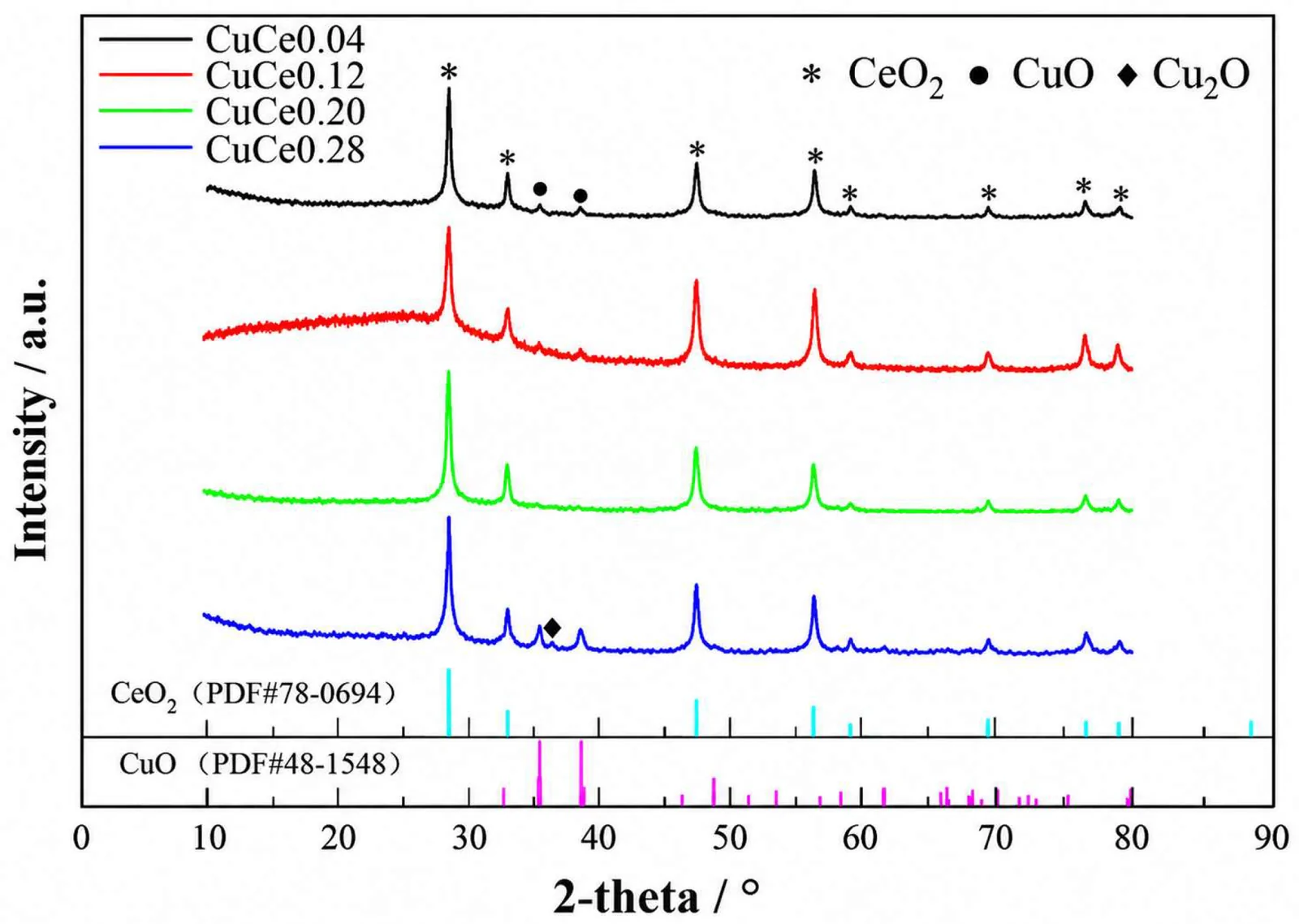
XRD patterns of the CuO-CeO_2_ catalysts.

The XRD patterns in Figure 2 reveal that precursor concentration also regulates the crystallization and distribution of Cu species at a fixed Cu loading. All samples show the characteristic fluorite CeO_2_ reflections at 28.5°, 33.1°, 47.5°, 56.3°, 59.1°, 69.4°, and 76.7°. Slight variations in the CeO_2_ diffraction peak positions are observed among the catalysts; the corresponding lattice parameters are quantified below using Bragg’s law (Table 1). Such small peak shifts may reflect changes in the CeO_2_ lattice environment but do not, by themselves, establish Cu species incorporation or solid-solution formation [17,22,29]. More importantly, the intensity of crystalline CuO/Cu_2_O signals varies markedly with precursor concentration, indicating that even with the same nominal Cu content, the distribution, dispersion, and crystallization of Cu-containing species can be influenced by precursor concentration during SSFP.

For CuCe0.20, no obvious crystalline CuO diffraction peaks are observed, suggesting that an intermediate precursor concentration provides a favorable flame environment for stabilizing Cu-containing species in highly dispersed or amorphous forms and/or allowing partial incorporation into the CeO_2_ lattice. In contrast, CuCe0.04, CuCe0.12, and CuCe0.28 show distinct copper oxide reflections, indicating partial copper oxide segregation. Therefore, the combined lattice-parameter and phase-analysis results suggest that precursor concentration influences the CeO_2_ lattice environment and the distribution of Cu-containing species during flame-phase particle formation and crystallization; XRD alone cannot conclusively determine the extent of Cu incorporation [30,31].

To quantify the small peak-position differences, the apparent lattice constants of the cubic fluorite CeO_2_ phase were estimated from the fitted (111), (200), (220), and (311) reflections using Bragg’s law (Cu Kα, λ = 1.5406 Å), and the unit cell volumes were calculated as V = a^3^. The values in Table 1 show slight, non-monotonic variations among the catalysts (a = 5.4037 − 5.4103 Å). These differences should be interpreted cautiously because the values are based on peak-position analysis rather than full-pattern refinement, and no instrumental zero-shift correction was available. In particular, the lattice parameters do not provide a direct measure of Cu incorporation or explain the catalytic activity ranking by themselves.

The crystallite sizes estimated from the CeO_2_ diffraction peaks using the Scherrer equation were 30, 16, 26, and 28 nm for CuCe0.04, CuCe0.12, CuCe0.20, and CuCe0.28, respectively. These XRD-derived values are subsequently considered together with the textural data only for comparison of particle-growth trends.

**Figure 3:**
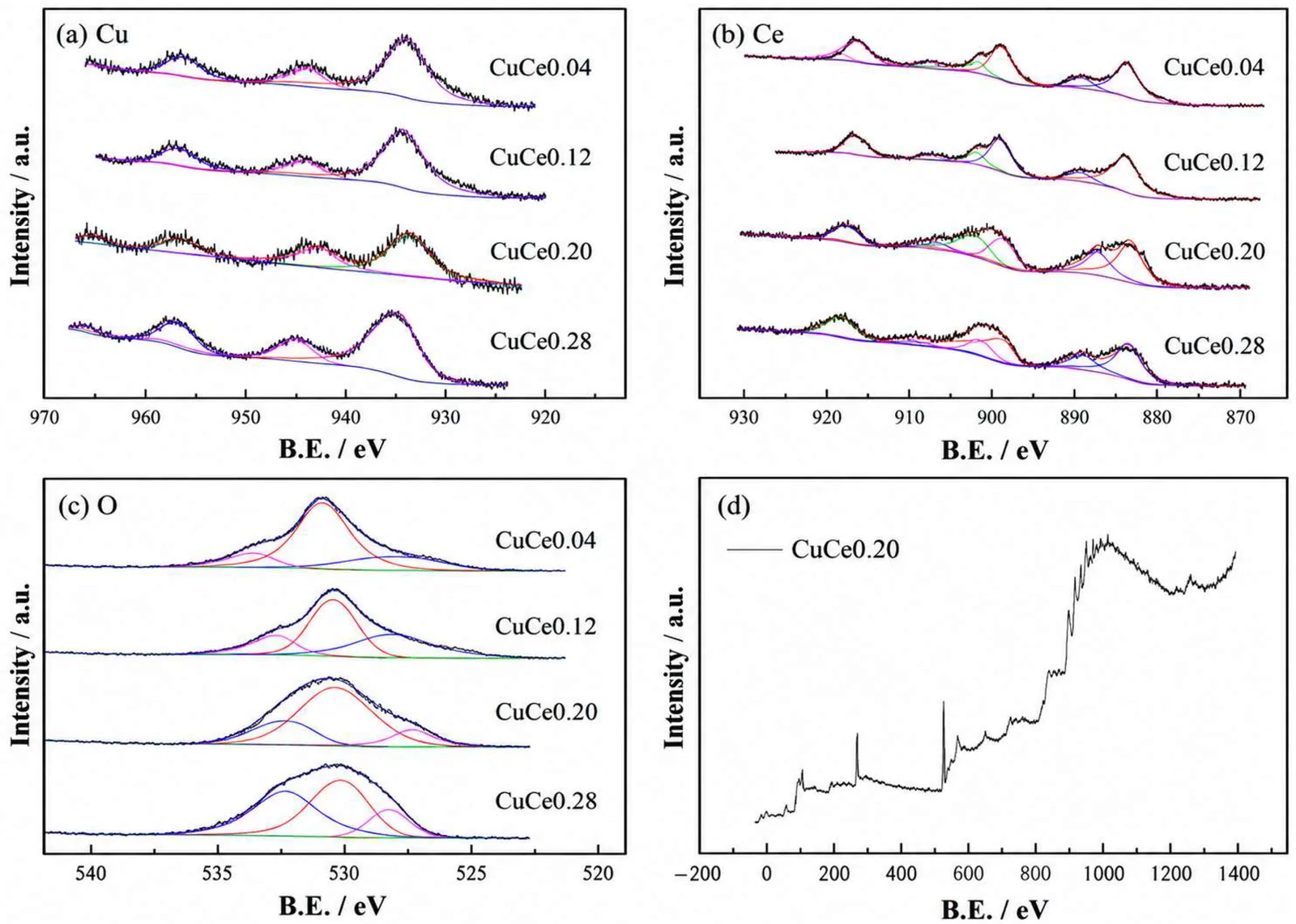
The XPS profiles of the CuO-CeO_2_ catalysts: (**a**) Cu 2p, (**b**) Ce 3d, (**c**) O 1s, and (**d**) survey spectra. In panels (**a**–**c**), the black lines represent the experimental spectra, while the colored lines indicate the overall fitted curves, individual deconvoluted peak components, and fitted backgrounds.

XPS was further used to examine the surface chemical states and oxygen species of the catalysts, as shown in Figure 3. The Cu 2p spectra indicate that Cu^2+^ is the predominant surface copper species, as supported by the characteristic shake-up satellite features. A lower-binding-energy Cu contribution is also observed; however, Cu^+^ and Cu^0^ cannot be unambiguously distinguished from the Cu 2p spectra alone because of their overlapping binding energies. Therefore, this contribution is denoted as Cu^+^/Cu^0^-related species rather than being assigned exclusively to Cu^+^. The relative fraction of this unresolved contribution decreases from 18.88% to 14.04% as the precursor concentration increases from 0.04 to 0.28 mol·L^−1^. Similarly, the Ce^3+^/Ce_total ratios decrease from 16.83% to 10.21%. These results suggest that the surface chemical states are sensitive to precursor-concentration-induced changes in the Cu-Ce environment. The surface Cu/Ce atomic ratios are 1.78, 2.18, 1.41, and 2.54 for CuCe0.04, CuCe0.12, CuCe0.20, and CuCe0.28, respectively, which are higher than the nominal bulk ratio. This confirms surface enrichment of Cu species, while the relatively lower Cu/Ce ratio in CuCe0.20, together with the absence of obvious crystalline CuO peaks, suggests more effective Cu species distribution or dispersion.

The O 1s spectra further demonstrate that precursor concentration changes the surface oxygen chemistry of CuO-CeO_2_ catalysts [30]. The deconvoluted O 1s spectra reveal three oxygen species, namely lattice oxygen (O_latt_), adsorbed oxygen species (O_ads_), and surface hydroxyl oxygen (O_oh_). As summarized in Table 2, CuCe0.04 and CuCe0.12 exhibit higher O_ads_ fractions, whereas CuCe0.20 shows the highest O_latt_ content while retaining a considerable amount of O_ads_. Therefore, the catalytic behavior cannot be simply correlated with the relative O_ads_ fraction alone. Instead, CuCe0.20 provides a more favorable balance between lattice oxygen storage and surface reactive oxygen availability.

This oxygen distribution is particularly important for C_3_H_6_ catalytic combustion. Surface reactive oxygen species can participate in low-temperature oxidation, while lattice oxygen can act as an oxygen reservoir to support oxygen migration and regeneration during continuous reaction. The XPS results therefore suggest that an intermediate precursor concentration does not merely increase one specific oxygen species but optimizes the balance between oxygen storage and oxygen availability.

**Figure 4:**
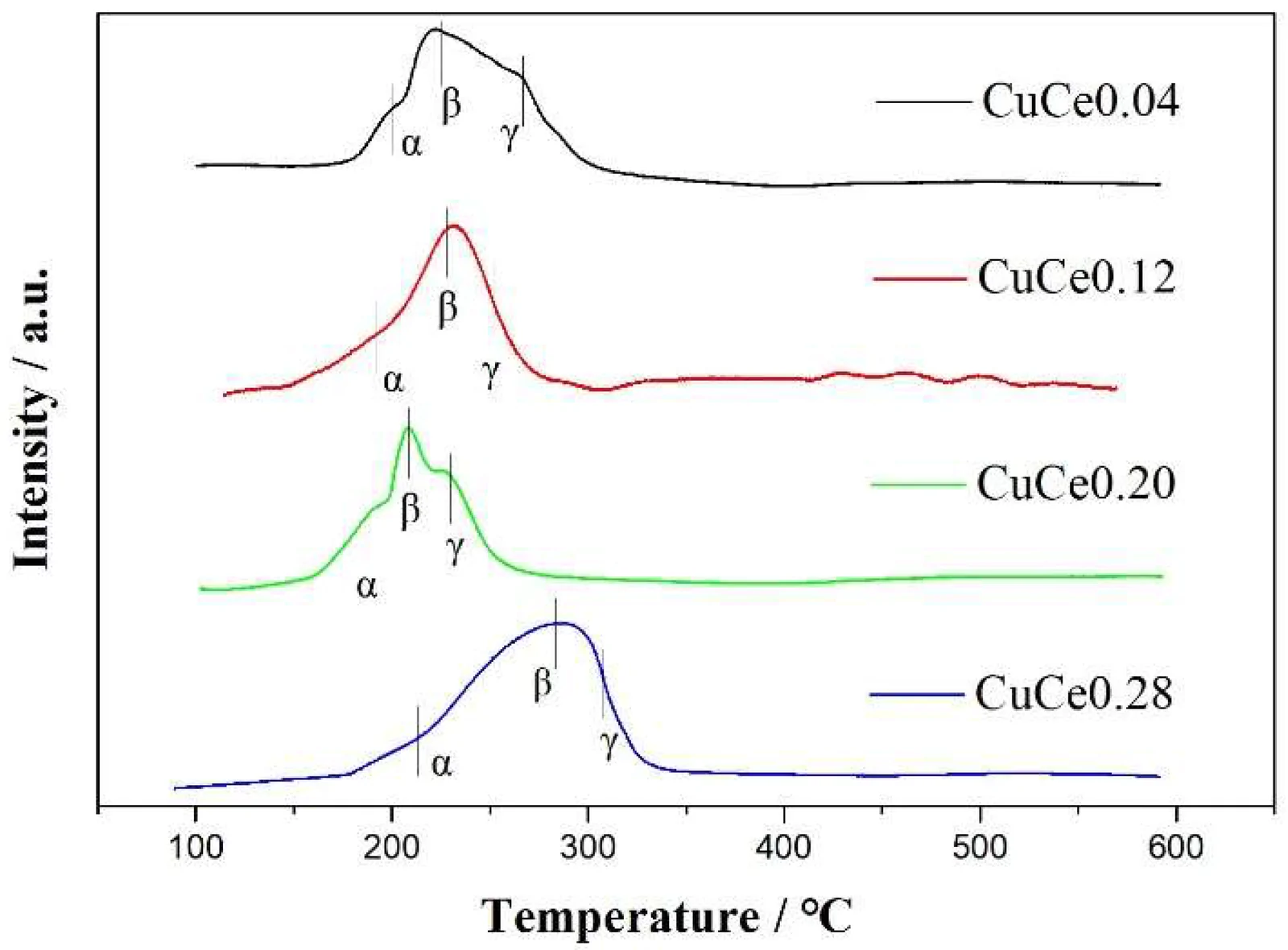
H_2_-TPR curves of CuO-CeO_2_ catalysts.

H_2_-TPR was further used to evaluate whether precursor concentration affects the redox behavior of CuO-CeO_2_ catalysts at the same Cu loading. As shown in Figure 4, all samples exhibit reduction peaks in the low-temperature region, indicating the presence of reducible CuCeX species and Cu species interacting with the CeO_2_ matrix. The α peak can be associated with highly dispersed or amorphous CuCeX species, the β peak with Cu species strongly interacting with or incorporated into the CeO_2_ lattice, and the γ peak with relatively larger CuO crystallites [32]. Notably, CuCe0.20 shows reduction peaks shifted to lower temperatures compared with the other samples, suggesting more favorable low-temperature oxygen activation and migration.

CuCe0.20 shows reduction features shifted toward lower temperatures compared with the other catalysts, indicating comparatively favorable low-temperature reducibility. The archived thermal conductivity detector (TCD) profiles do not retain the calibration factor required for absolute H_2_-uptake quantification; therefore, the present H_2_-TPR results are interpreted comparatively rather than as a quantitative measure of oxygen storage capacity (OSC) or Cu reduction degree.

As a stoichiometric reference, complete reduction of the nominal Cu^2+^ species at Cu/(Cu + Ce) = 35 mol.% would require approximately 2.505 mmol H_2_ g^−1^ catalyst, assuming an ideal 0.35CuO-0.65CeO_2_ composition. However, this value cannot be directly used to determine the Cu reduction degree from the present profiles because Ce-related reduction and reducible interfacial oxygen may also contribute to H_2_ consumption.

Together with the XRD and XPS results, the lower-temperature reduction features of CuCe0.20 suggest that the intermediate precursor concentration favors the formation of Cu-Ce-O species with enhanced low-temperature redox response [32,33].

### 3.2. Textural and Morphological Evolution During SSFP

At a fixed Cu/(Cu + Ce) molar ratio of 35 mol.%, changing the total metallic precursor concentration directly modifies the solute loading of atomized droplets and the particle-formation environment in the self-sustained flame. Therefore, differences among the CuCeX samples should be interpreted as the consequence of precursor-concentration-driven nucleation, collision, aggregation, and crystallite growth, rather than changes in metal composition.

The N_2_ adsorption-desorption and SEM analyses are used here to describe textural and morphological characteristics; phase composition and surface chemical states are interpreted from the XRD and XPS results discussed above.

**Figure 5:**
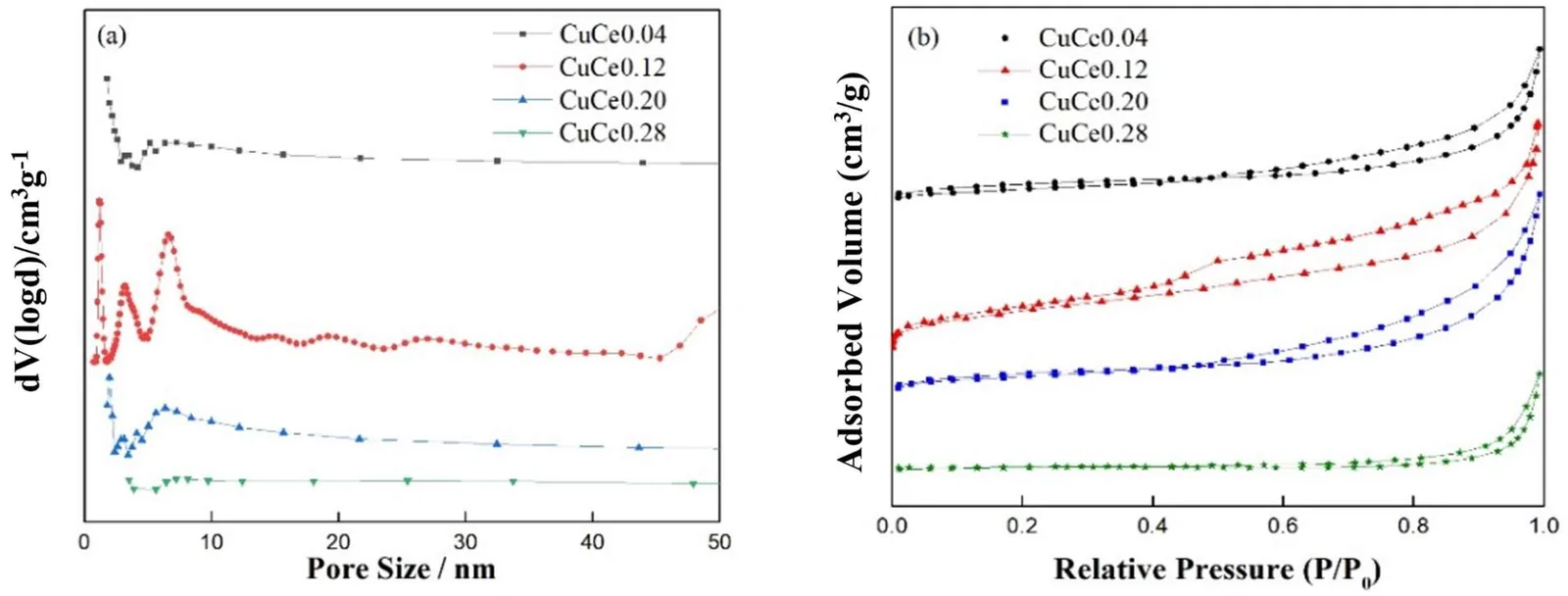
(**a**) Apparent pore-size distributions derived from N_2_ desorption data using the BJH method and (**b**) N_2_ adsorption-desorption isotherms of the prepared catalysts.

The textural properties of the CuO-CeO_2_ catalysts are summarized in Table 3, and the corresponding N_2_ adsorption-desorption results are shown in Figure 5. As the precursor concentration increased from 0.12 to 0.28 mol·L^−1^, the specific surface area decreased from 29.8 to 5.3 m^2^·g^−1^, indicating that higher precursor concentration promotes particle collision and aggregation during SSFP. CuCe0.04 also exhibited a relatively low surface area, suggesting that excessively dilute precursor conditions may likewise be unfavorable for maintaining a highly accessible surface structure.

Although CuCe0.12 possessed the highest surface area, this textural advantage did not directly translate into the best catalytic performance. These textural characteristics alone cannot account for the activity trend and should therefore be interpreted together with the phase, surface-chemical, and redox characteristics discussed above. Thus, precursor concentration should be considered a process parameter that governs multiple catalyst properties simultaneously.

**Figure 6:**
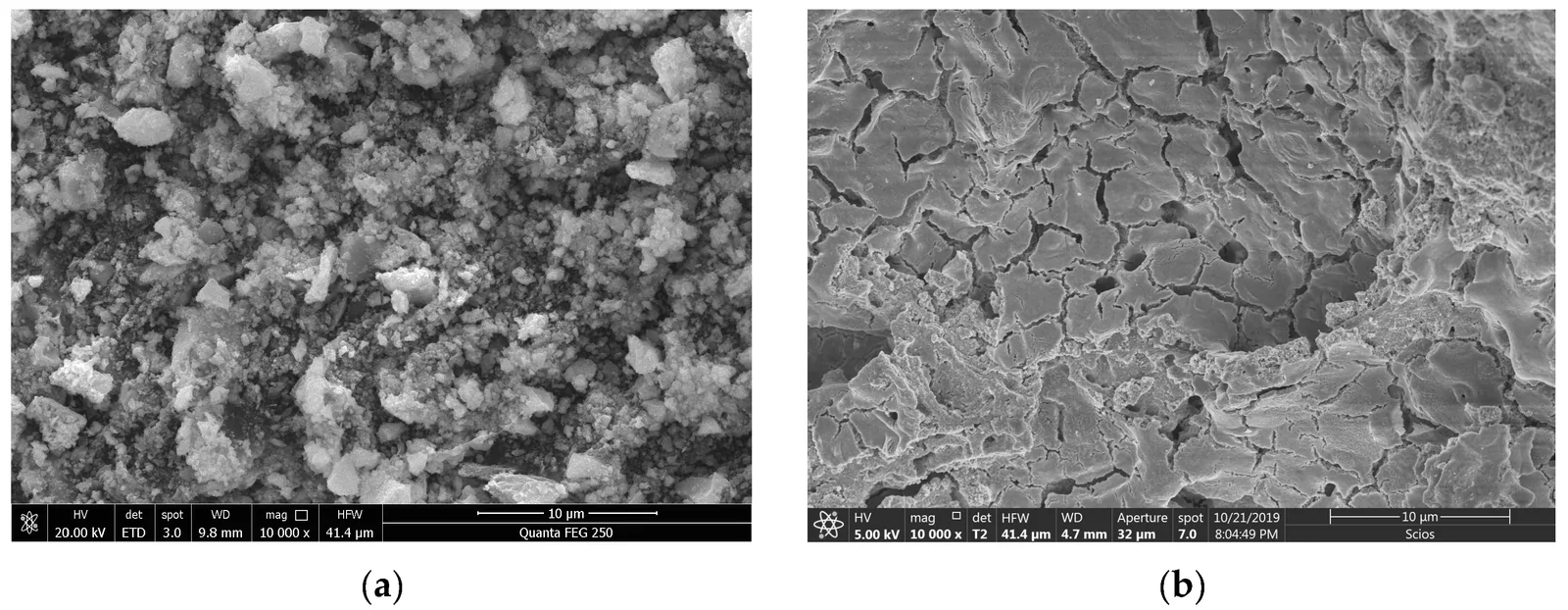

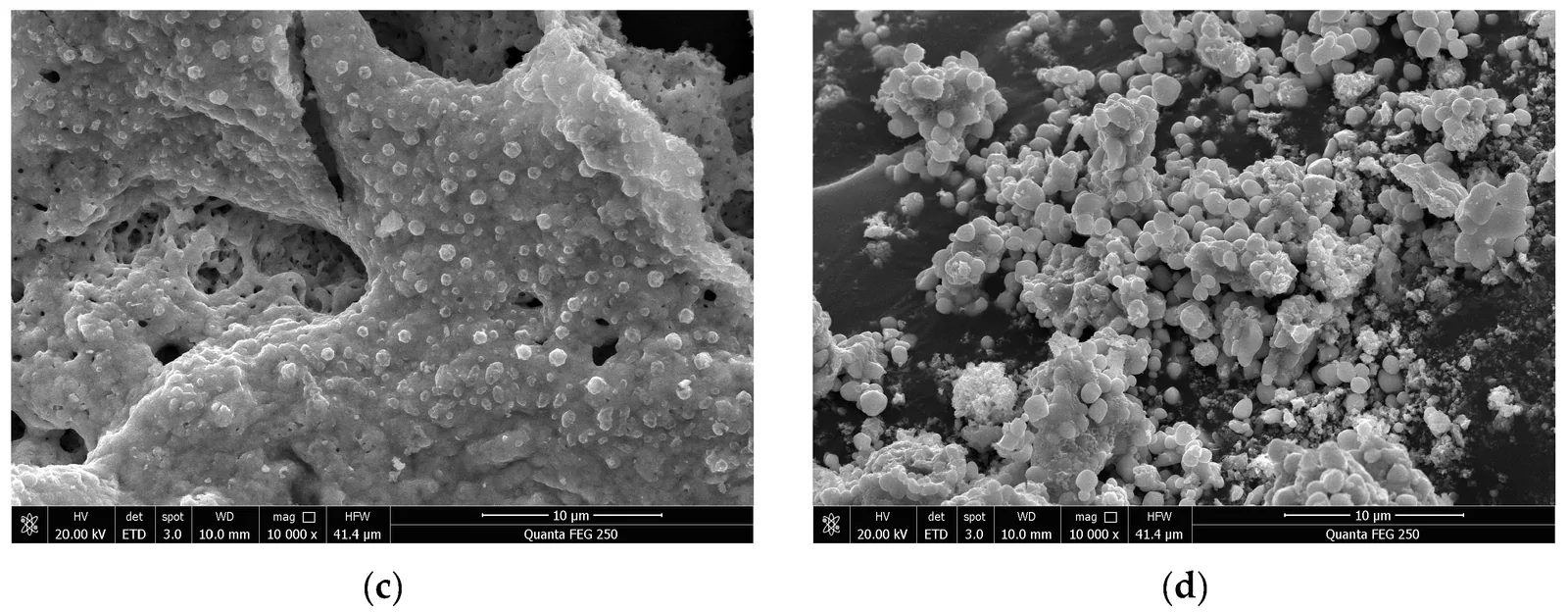
SEM images of CuO-CeO_2_ composites: (**a**) CuCe0.04, (**b**) CuCe0.12, (**c**) CuCe0.20, (**d**) CuCe0.28.

The SEM images in Figure 6 further support the role of precursor concentration in governing flame-derived particle assembly. CuCe0.04 consists of irregular particles, indicating insufficient structural development at low precursor concentration. When the precursor concentration increased to 0.12 mol·L^−1^, a more continuous aggregate structure with visible interparticle voids was formed. Further increasing the concentration to 0.20 and 0.28 mol·L^−1^ preserved the bulk morphology but generated numerous microsphere-like particles on the surface. Since the Cu loading and gas-liquid feeding conditions were kept constant, these morphological changes mainly originate from the altered precursor density in the flame, which changes droplet-to-particle conversion, particle collision frequency, and aggregation behavior during SSFP.

### 3.3. C_3_H_6_ Catalytic Combustion Performance Beyond Composition Optimization

**Figure 7:**
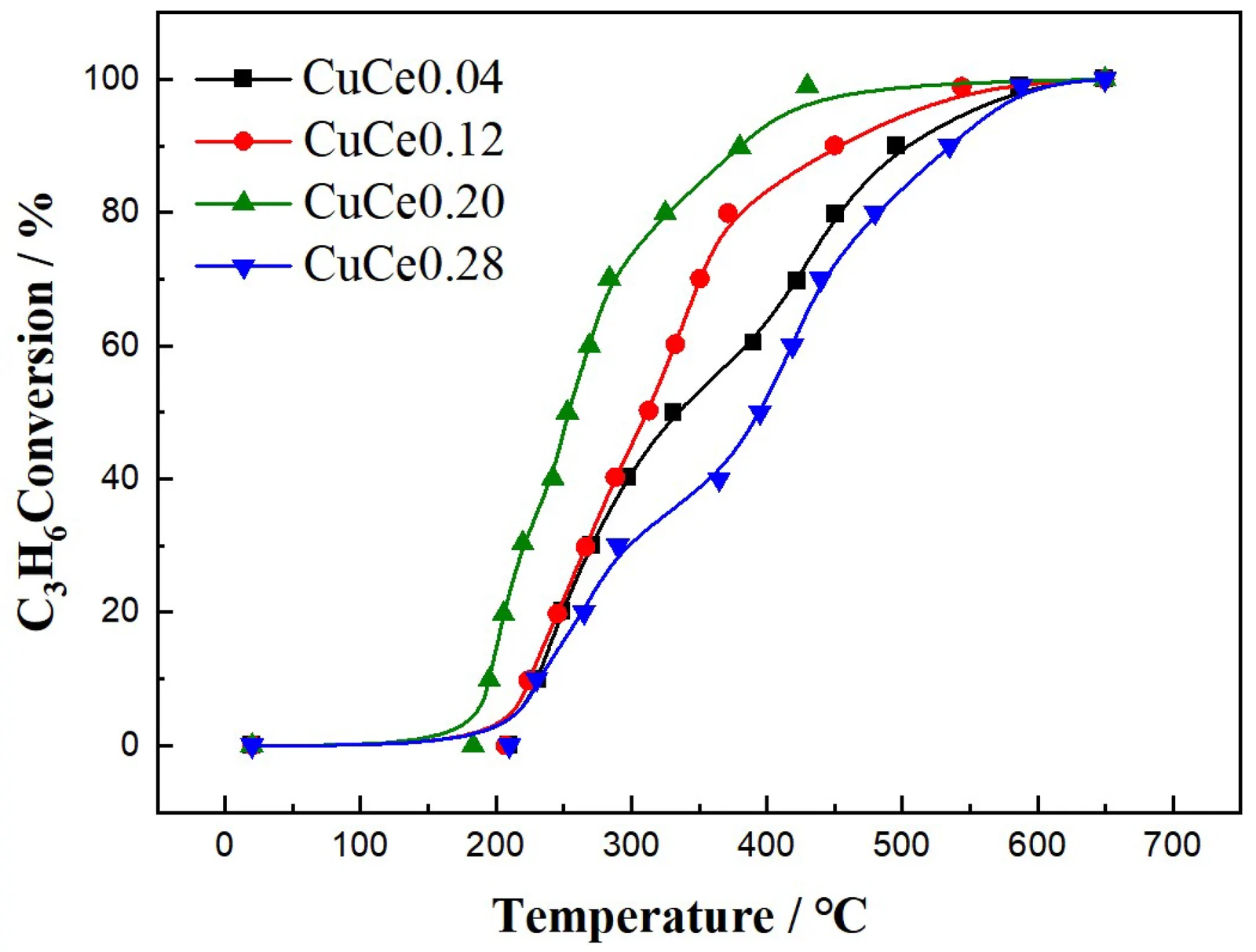
C_3_H_6_ conversion over different CuO-CeO_2_ catalysts.

The catalytic performance of the CuO-CeO_2_ catalysts was evaluated to verify whether precursor-concentration engineering can effectively tune C_3_H_6_ combustion activity at a fixed Cu loading. As shown in Figure 7 and Table 4, activity follows the order of CuCe0.28 < CuCe0.04 < CuCe0.12 < CuCe0.20, confirming that precursor concentration has a pronounced influence on catalytic performance even when the Cu/(Cu + Ce) molar ratio remains unchanged.

The temperatures corresponding to 50%, 80%, and 90% C_3_H_6_ conversion are denoted as T_50_, T_80_, and T_90_, respectively. CuCe0.20 exhibited the highest catalytic activity, with corresponding values of 253, 325, and 380 °C, and achieved nearly complete C_3_H_6_ conversion at 450 °C. The characteristic conversion temperatures of CuCe0.20 are consistent with those obtained in our previous parallel tests for a catalyst prepared at the same nominal Cu/(Cu + Ce) ratio and precursor concentration [31], further supporting the reproducibility of the observed catalytic performance. It should be emphasized that this high activity was obtained by optimizing precursor concentration rather than changing Cu loading. This demonstrates that precursor concentration is not a secondary preparation variable but an effective process parameter capable of tuning catalytic performance to a level comparable to composition optimization.

To provide an additional quantitative assessment of catalytic performance, apparent mass-specific C_3_H_6_ consumption rates were estimated from the available low-conversion data, as summarized in Table 5. The four catalysts exhibited comparable rates of 7.17–7.36 mmol·g^−1^·h^−1^ at approximately 10% conversion. Notably, CuCe0.20 reached this conversion level at 195.20 °C, whereas the other catalysts required temperatures of 223.80–230.86 °C, consistent with their lower characteristic conversion temperatures. Because the rates were obtained at different reaction temperatures, only one non-zero low-conversion data point was available for each catalyst, and mass/heat transfer limitations could not be independently quantified, the present results cannot establish differences in intrinsic reaction kinetics or support a reliable Arrhenius determination of the apparent activation energy.

The superior activity of CuCe0.20 can be attributed to the combined effects of precursor-concentration-regulated structure and surface chemistry. Although CuCe0.12 possesses the highest surface area and the smallest crystallite size, its activity is lower than that of CuCe0.20, indicating that textural properties alone cannot determine C_3_H_6_ combustion performance. Conversely, the poor activity of CuCe0.28 is related to its significantly reduced surface area and partial Cu oxide segregation, which may limit gas-solid contact and effective redox cycling. Therefore, the optimized performance of CuCe0.20 originates from the balance among textural characteristics, Cu species distribution/dispersion, oxygen storage and availability, and low-temperature reducibility.

Compared with noble-metal catalysts, CuCe0.20 exhibits competitive C_3_H_6_ combustion activity, achieving 90% conversion at 380 °C (T_50_ = 253 °C), compared with 450 °C for 1 wt.% Au/Al_2_O_3_ [34] and 95% conversion at 417 °C for 4.1 wt.% Au/Al_2_O_3_ [35]. Transition metal oxides, including Co_3_O_4_-based [8] and Mn-based catalysts [36,37], have also demonstrated promising propene oxidation performance. Although differences in reaction conditions limit direct comparisons, these studies highlight the importance of catalyst structure and redox properties. The present work demonstrates that precursor-concentration engineering offers an effective strategy for enhancing CuO-CeO_2_ catalytic activity without altering the Cu/Ce ratio.

Compared with our previous composition-optimized CuO-CeO_2_ catalyst [31], the optimized CuCe0.20 in this work reaches a similar T_90_ value of 380 °C while using a different regulation strategy. This indicates that precursor-concentration engineering can achieve high catalytic activity without further changing the Cu/Ce ratio. More importantly, the present work provides additional insight into how precursor concentration affects catalyst formation, oxygen species distribution, and post-reaction oxygen-species evolution during SSFP.

**Figure 8:**
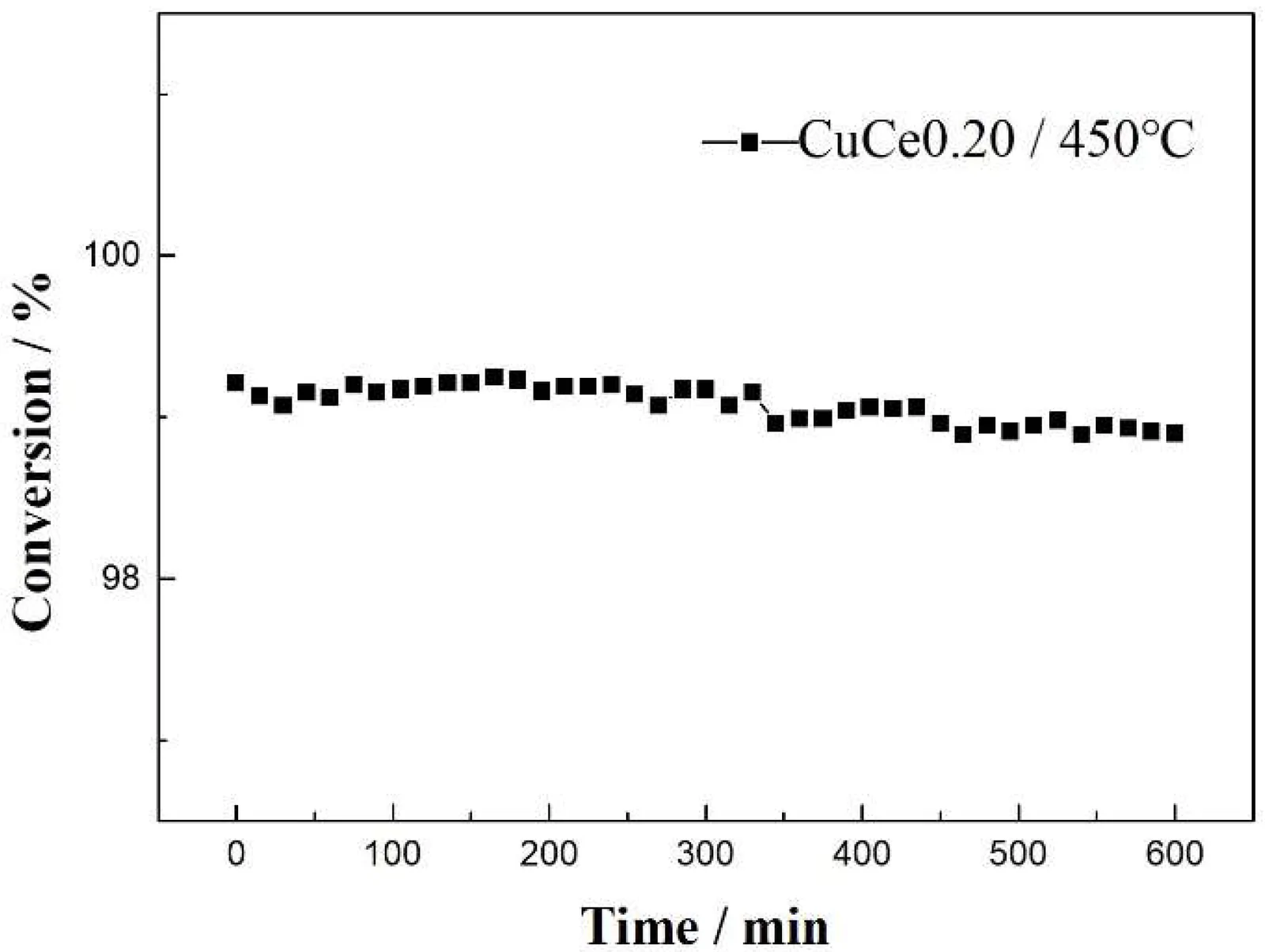
The C_3_H_6_ conversion during a 600-min long-term catalytic combustion test.

Considering its optimal catalytic activity, CuCe0.20 was further evaluated in a 600 min stability test at 450 °C. As shown in Figure 8, C_3_H_6_ conversion remained stable throughout the test, and the average conversion in the last 60 min decreased by only 0.2% compared with that during the first 60 min. In addition, CuCe0.20 exhibited an apparent CO_2_ selectivity of 99.9% based on the measured dry-gas concentrations. This high apparent value indicates that CO_2_ was the predominant detected oxidation product under the tested conditions, although a rigorous carbon balance could not be established because the outlet molar flow rate and all possible oxygenated byproducts were not independently quantified. These results demonstrate that the optimized precursor concentration promotes C_3_H_6_ conversion while maintaining CO_2_ as the predominant detected oxidation product under continuous operation.

### 3.4. Post-Reaction Oxygen-Species Evolution and Structural Stability

Practical VOC combustion catalysts must maintain both stable oxidation performance and favorable redox characteristics under continuous operation. Therefore, the optimized CuCe0.20 catalyst was further examined after the 600 min stability test to clarify the post-reaction evolution of its textural characteristics, crystalline phase, surface oxygen species, and redox properties.

**Figure 9:**
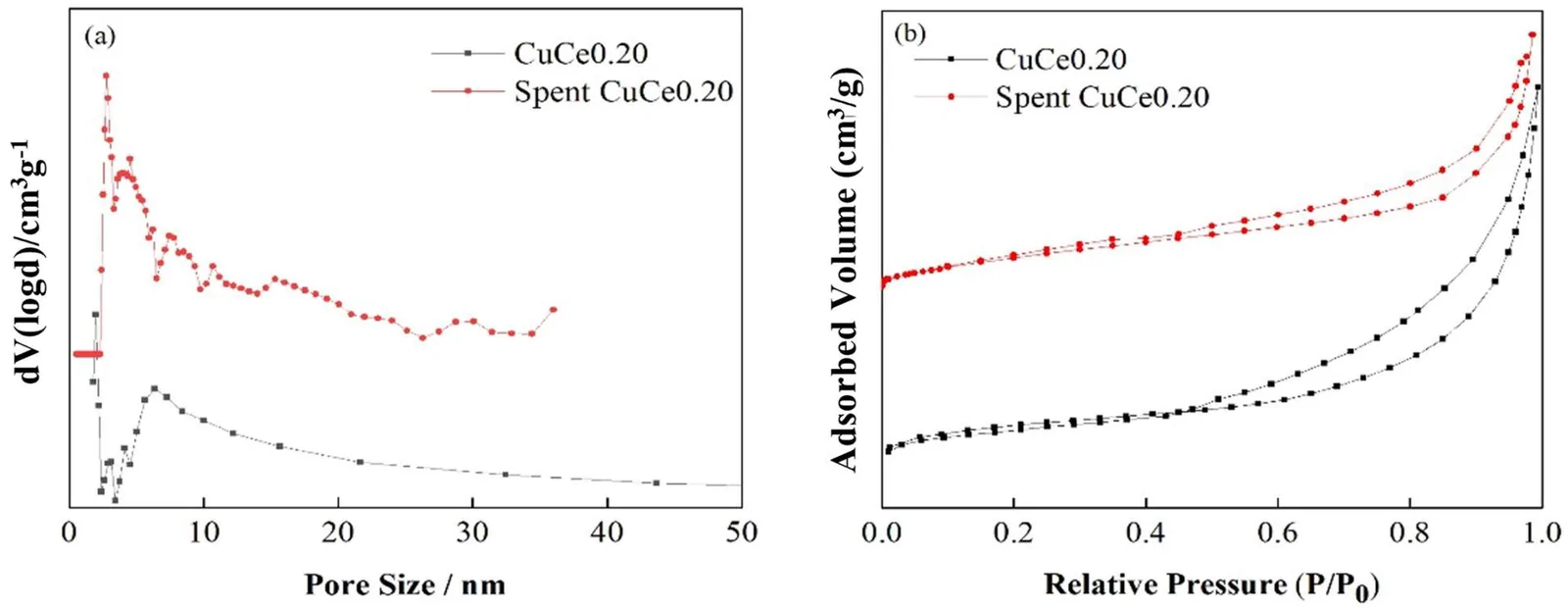
(**a**) Apparent pore-size distribution calculated from the desorption branch of the prepared samples; (**b**) N_2_ adsorption-desorption isotherms.

Figure 9 compares the apparent BJH-derived pore-size distributions and N_2_ adsorption-desorption isotherms of fresh and spent CuCe0.20. Noticeable changes after the stability test indicate textural evolution during long-term high-temperature reaction. The quantitative textural data in Table 6 show that the specific surface area decreases from 25.8 to 12.4 m^2^·g^−1^ and the pore volume decreases from 0.05 to 0.03 cm^3^(STP)·g^−1^, while the crystallite size increases from 26 to 29 nm. These changes indicate moderate textural degradation, sintering, and crystallite growth during the long-term reaction.

Despite substantial surface area loss, CuCe0.20 maintained stable C_3_H_6_ conversion, suggesting that catalytic stability is not solely determined by textural properties. This behavior may be attributed to the preservation of the CeO_2_ framework, retained low-temperature reducibility, and redistribution of surface oxygen species, as further supported by the following XRD, XPS, and H_2_-TPR analyses.

**Figure 10:**
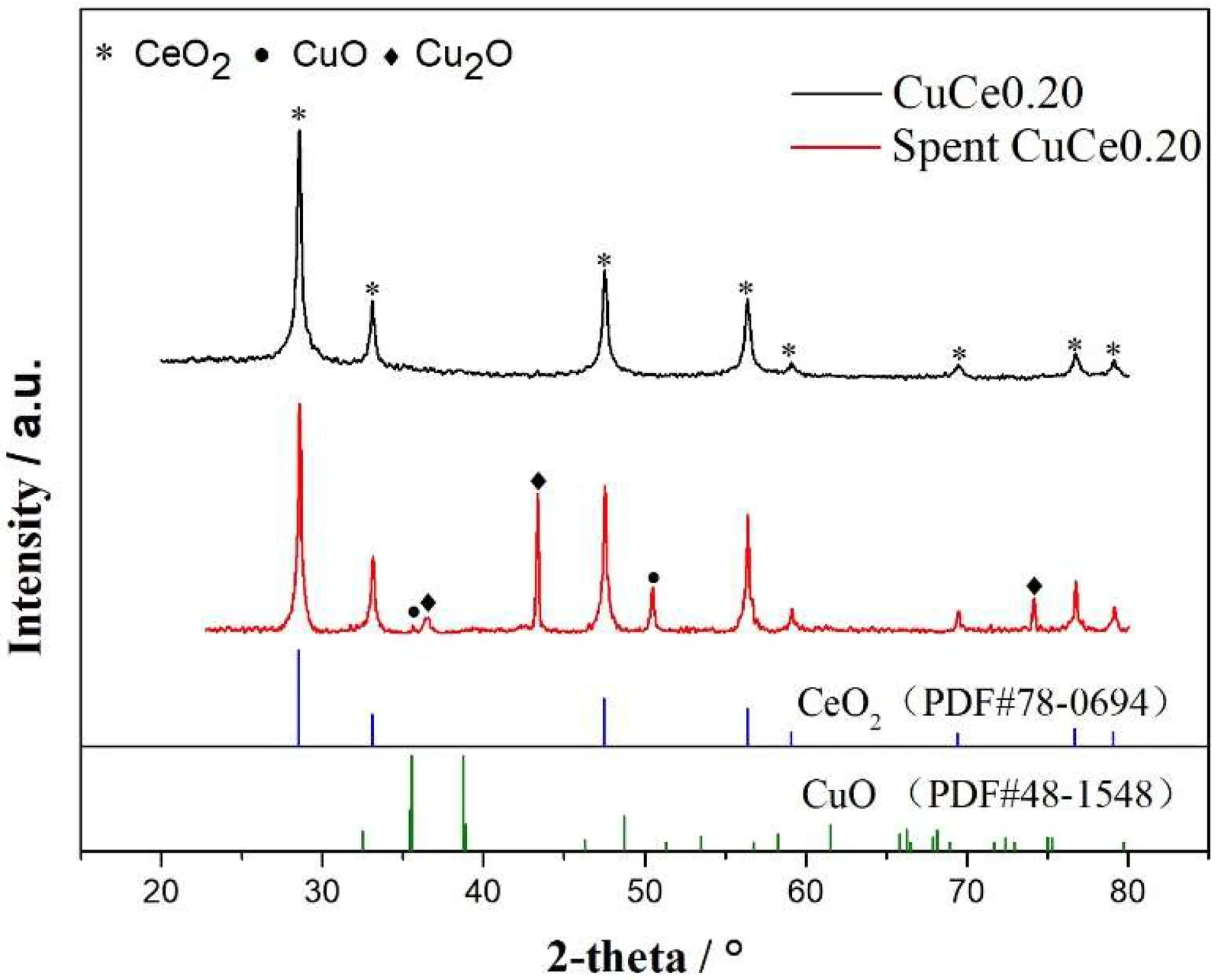
XRD patterns of fresh and spent CuCe0.20.

The XRD patterns of fresh and spent CuCe0.20 are compared in Figure 10. The characteristic fluorite CeO_2_ reflections are retained after the 600 min reaction, indicating that the CeO_2_ framework is largely preserved. Meanwhile, additional or intensified diffraction peaks assigned to CuO and Cu_2_O are observed for the spent catalyst, suggesting that part of the initially highly dispersed or amorphous CuCeX species crystallized during the high-temperature reaction. Although partial CuCeX crystallization occurs, the preserved CeO_2_ framework indicates that the main CuO-CeO_2_ structure remains sufficiently stable, which is consistent with the stable catalytic performance.

**Figure 11:**
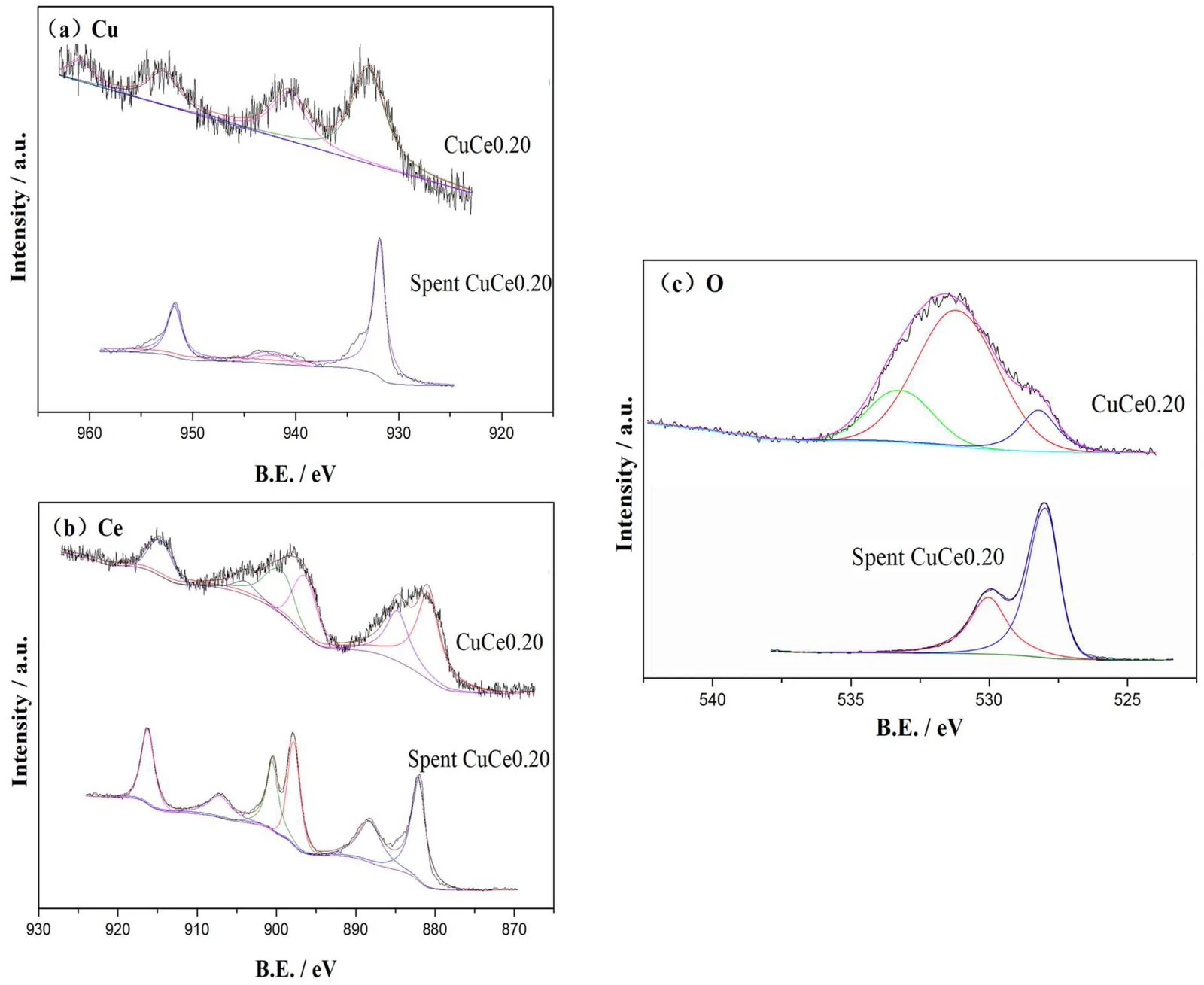
XPS spectra of fresh and spent CuCe0.20 catalysts: (**a**) Cu 2p, (**b**) Ce 3d, and (**c**) O 1s. The black/gray lines represent the experimental spectra, the magenta lines indicate the overall fitted spectra, and the other colored lines represent the deconvoluted peak components and fitted backgrounds.

Figure 11 shows the XPS spectra of fresh and spent CuCe0.20. After the stability test, the Cu- and Ce-related spectra still retain their characteristic features, indicating that the main Cu and Ce species are preserved during long-term catalytic combustion. However, the sharper and more intense peaks observed for spent CuCe0.20 suggest changes in the local surface environment, which are consistent with the crystallization of initially amorphous or highly dispersed CuCeX species observed by XRD.

More pronounced changes are observed in the O 1s spectra. As summarized in Table 7, the relative O_latt_ fraction increases from 38% to 64%, whereas O_ads_ and O_oh_ decrease after the reaction. The substantial reduction in O_oh_ may result from the dehydration of surface hydroxyl groups under high-temperature conditions, contributing to the relative increase in O_latt_. Meanwhile, the XRD and H_2_-TPR results indicate partial crystallization of highly dispersed or amorphous CuCeX species, suggesting that surface structural reorganization may also contribute to the altered oxygen distribution. Therefore, the increased O_latt_ fraction likely reflects the combined effects of oxygen species redistribution and CuCeX structural evolution, rather than a proportional increase in absolute lattice oxygen content.

**Figure 12:**
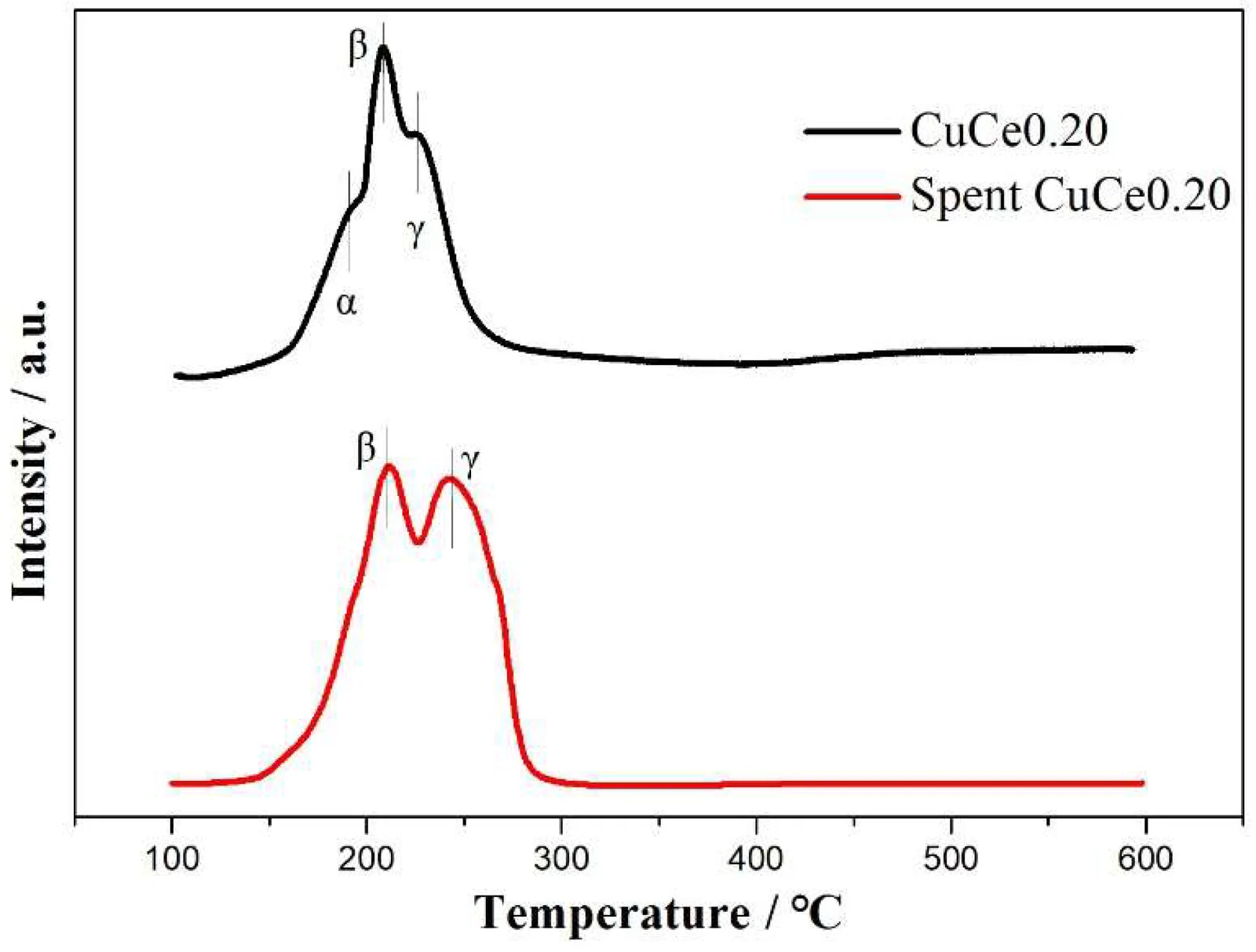
H_2_-TPR profiles of fresh and spent CuCe0.20 catalysts.

The H_2_-TPR profiles of fresh and spent CuCe0.20 are shown in Figure 12. For the fresh catalyst, the reduction peaks correspond to highly dispersed/amorphous CuCeX species, Cu species strongly interacting with the CeO_2_ lattice, and relatively larger CuO crystallites. After the long-term reaction, the α peak associated with highly dispersed or amorphous CuCeX species becomes much less evident, while the γ peak assigned to larger CuO crystallites becomes more pronounced. This result further confirms partial crystallization of the initially dispersed CuCeX species during the reaction.

Importantly, pronounced reduction features remain in the low-temperature region for spent CuCe0.20, indicating that a substantial fraction of its low-temperature reducibility is retained after reaction. Because absolute H_2_ uptake could not be reconstructed from the archived TCD profiles, this comparison is restricted to the relative evolution of reduction behavior rather than quantitative OSC. Combined with the XRD and XPS results, the stable catalytic performance can therefore be associated with the largely preserved CeO_2_ framework, persistent Cu-Ce interaction, redistribution of surface oxygen species, and retained low-temperature reducibility.

Overall, the results demonstrate that precursor concentration acts as an independent process parameter during SSFP, regulating particle formation, Cu-species distribution, surface oxygen chemistry, and low-temperature reducibility at a fixed Cu loading. The optimized precursor concentration of 0.20 mol·L^−1^ provides a favorable balance among structural accessibility, Cu-Ce interaction, oxygen-species distribution, and redox behavior, thereby enabling efficient and stable C_3_H_6_ catalytic combustion.

## 4. Conclusions

In this work, precursor concentration was identified as an effective process parameter for regulating the formation of CuO-CeO_2_ mixed-oxide catalysts during self-sustained flame pyrolysis. Different from previous composition-regulation studies, the Cu/(Cu + Ce) molar ratio was fixed at 35 mol.% and the total metallic precursor concentration was varied from 0.04 to 0.28 mol·L^−1^. This design decouples the effect of precursor concentration from that of metal composition and provides a process-oriented understanding of how precursor density in the flame governs catalyst formation.

The results demonstrate that precursor concentration significantly affects the flame-derived structure of CuO-CeO_2_ catalysts, including particle aggregation, crystallite growth, Cu species distribution, oxygen species distribution, and redox behavior. At excessively low or high precursor concentrations, the catalyst structure is less favorable due to poor particle regulation or enhanced particle aggregation. In contrast, the intermediate precursor concentration of 0.20 mol·L^−1^ provides a suitable flame environment for forming CuCe0.20 with balanced textural properties, favorable Cu-Ce interaction, optimized oxygen species distribution, and improved low-temperature reducibility.

The optimized CuCe0.20 catalyst exhibited the best catalytic performance for C_3_H_6_ combustion, achieving 90% conversion at 380 °C and nearly complete conversion at 450 °C. Importantly, this high activity was achieved by precursor-concentration regulation rather than further changing the Cu/Ce composition, confirming that precursor concentration is an independent and effective tuning parameter for SSFP-derived CuO-CeO_2_ catalysts. The catalyst also maintained stable activity during a 600 min test at 450 °C with an apparent CO_2_ selectivity of 99.9%, indicating that CO_2_ remained the predominant detected oxidation product during continuous operation. Post-reaction characterization further revealed the origin of this stability. Although partial crystallization of initially highly dispersed or amorphous CuCeX species occurred during the long-term reaction, the CeO_2_ framework and Cu-Ce interaction were largely retained. Meanwhile, the increased relative lattice-oxygen fraction in the spent catalyst indicates a redistribution of surface oxygen species during long-term reaction; this relative change should not be interpreted as a direct quantitative increase in absolute oxygen storage capacity. Therefore, the stable catalytic performance of CuCe0.20 can be attributed to the combined effects of retained structural integrity, persistent Cu-Ce interaction, redistribution of surface oxygen species, and retained low-temperature reducibility.

Overall, this study extends the design strategy of flame-made CuO-CeO_2_ catalysts from metal-composition optimization to precursor-concentration engineering. The findings demonstrate that regulating precursor concentration during SSFP is a practical and effective approach for tailoring non-noble-metal mixed-oxide catalysts, providing useful guidance for scalable catalyst preparation and VOC catalytic combustion applications.

## Figures and Tables

**Table 1:** Lattice parameters of the cubic fluorite CeO_2_ phase estimated from XRD peak positions.

Sample	Lattice Constant, a (Å)	Unit Cell Volume, V (Å^3^)
CuCe0.04	5.4080	158.160
CuCe0.12	5.4103	158.371
CuCe0.20	5.4082	158.182
CuCe0.28	5.4037	157.786

**Table 2:** Relative contents of surface oxygen species obtained from O 1s deconvolution.

Sample	O_latt_/%	O_ads_/%	O_oh_/%
CuCe0.04	22.56	65.10	12.37
CuCe0.12	22.73	65.48	11.79
CuCe0.20	37.99	45.02	16.99
CuCe0.28	26.89	54.59	18.52

**Table 3:** Textural properties and XRD-derived crystallite sizes of the CuO-CeO_2_ catalysts.

Sample	BET Analysis		XRD Analysis
	S_BET_/m^2^·g^−1^	Vp/cm^3^(STP)·g^−1^	D ^a^/nm
CuCe0.04	16.3	0.04	30
CuCe0.12	29.8	0.04	16
CuCe0.20	25.8	0.05	26
CuCe0.28	5.3	0.03	28

^a^ Crystallite sizes were calculated from the CeO_2_ diffraction peaks using the Scherrer equation.

**Table 4:** T_50_, T_80_, and T_90_ values over the CuO-CeO_2_ catalysts.

Sample	T_50_/°C	T_80_/°C	T_90_/°C
CuCe0.04	331	451	496
CuCe0.12	312	371	445
CuCe0.20	253	325	380
CuCe0.28	395	480	535

**Table 5:** Low-conversion C_3_H_6_ consumption rates over the CuO-CeO_2_ catalysts.

Sample	Temperature °C	Conversion %	Apparent Specific Rate (mmol·g^−1^·h^−1^)
CuCe0.04	230.86	9.94	7.313
CuCe0.12	223.80	9.74	7.166
CuCe0.20	195.20	9.81	7.218
CuCe0.28	230.00	10.00	7.357

**Table 6:** Textural properties of fresh and spent CuCe0.20 catalysts.

Sample	BET Analysis		XRD Analysis
	S_BET_/m^2^·g^−1^	Vp/cm^3^(STP)·g^−1^	D/nm
CuCe0.20	25.8	0.05	26
Spent CuCe0.20	12.4	0.03	29

**Table 7:** Relative contents of oxygen species in fresh and spent CuCe0.20.

Sample	O_latt/_%	O_ads_/%	O_oh_/%
CuCe0.20	37.99	45.02	16.99
Spent CuCe0.20	63.80	34.08	2.12

## Data Availability

The original contributions presented in this study are included in the article. Further inquiries can be directed to the corresponding author.
